# FACT and FAIR with Big Data allows objectivity in science: The view of crystallography

**DOI:** 10.1063/1.5124439

**Published:** 2019-10-25

**Authors:** John R. Helliwell

**Affiliations:** Department of Chemistry, University of Manchester, Manchester M13 9PL, United Kingdom

## Abstract

A publication is an important narrative of the work done and interpretations made by researchers securing a scientific discovery. As The Royal Society neatly states though, “Nullius in verba” (“Take nobody's word for it”), whereby the role of the underpinning data is paramount. Therefore, the objectivity that preserving that data within the article provides is due to readers being able to check the calculation decisions of the authors. But how to achieve full data archiving? This is the raw data archiving challenge, in size and need for correct metadata. Processed diffraction data and final derived molecular coordinates archiving in crystallography have achieved an exemplary state of the art relative to most fields. One can credit IUCr with developing exemplary peer review procedures, of narrative, underpinning structure factors and coordinate data and validation report, through its checkcif development and submission system introduced for Acta Cryst. C and subsequently developed for its other chemistry journals. The crystallographic databases likewise have achieved amazing success and sustainability these last 50 years or so. The wider science data scene is celebrating the FAIR data accord, namely, that data be Findable, Accessible, Interoperable, and Reusable [Wilkinson *et al*., “Comment: The FAIR guiding principles for scientific data management and stewardship,” Sci. Data **3**, 160018 (2016)]. Some social scientists also emphasize more than FAIR being needed, the data should be “FACT,” which is an acronym meaning Fair, Accurate, Confidential, and Transparent [van der Aalst *et al*., “Responsible data science,” Bus Inf. Syst. Eng. **59**(5), 311–313 (2017)], this being the issue of ensuring reproducibility not just reusability. (Confidentiality of data not likely being relevant to our data obviously.) Acta Cryst. B, C, E, and IUCrData are the closest I know to being both FACT and FAIR where I repeat for due emphasis: the narrative, the automatic “general” validation checks, and the underpinning data are checked thoroughly by subject specialists (i.e., the specialist referees). IUCr Journals are also the best that I know of for encouraging and then expediting the citation of the DOI for a raw diffraction dataset in a publication; examples can be found in IUCrJ, Acta Cryst D, and Acta Cryst F. The wish for a checkcif for raw diffraction data has been championed by the IUCr Diffraction Data Deposition Working Group and its successor, the IUCr Committee on Data.

## I. INTRODUCTION

**FIG. 1.**
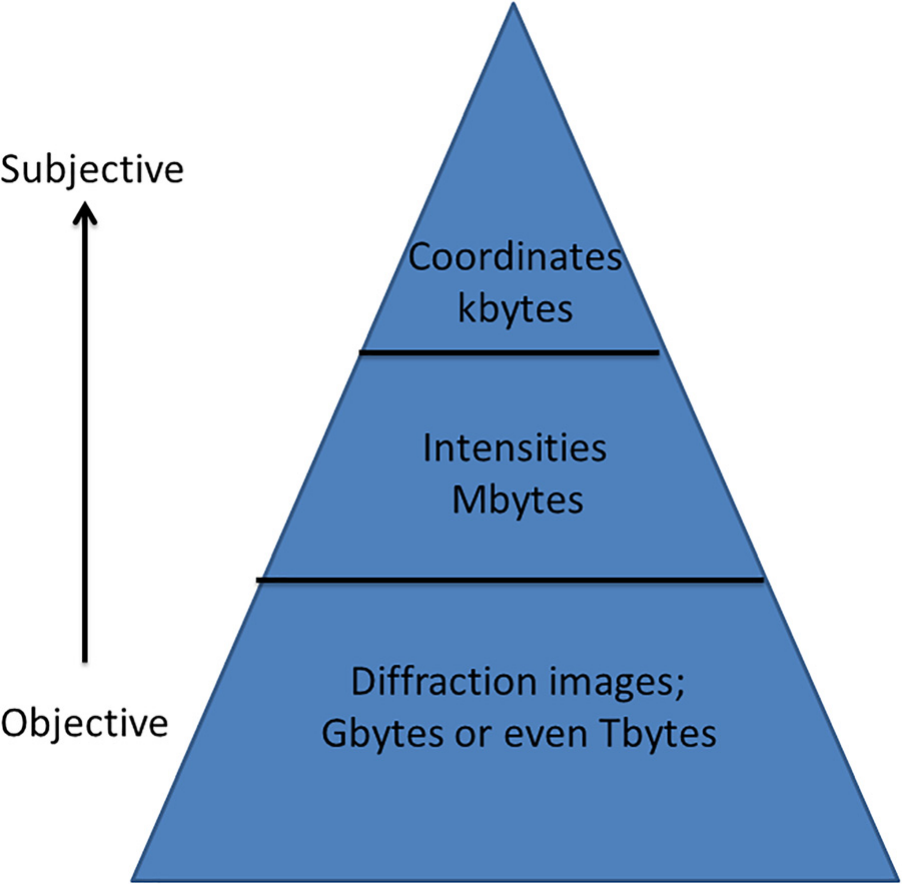
The Big Data pyramid labeled for typical crystallography dataset file sizes for raw diffraction images at the base, the processed structure factors in the middle, and the derived atomic coordinates and their respective atomic displacement parameters in the top of the pyramid.

Figure 1 shows a schematic representation of the scientific method, crystallography style, with our typical file sizes. Rather provocatively, perhaps I have labeled a direction of travel of the research from objectivity to subjectivity. What do I mean by that? To realize the very desirable goal of objectivity in science requires archiving of the primary experimental, raw, data. This is, however, the most burdensome in terms of sizes of data files. During processing of the raw diffraction data, decisions are made by the researcher and subjectivity is introduced. During model refinement, further decisions are made and more subjectivity is introduced in arriving at the final protein “model.” The article narrative describes those methods and decisions made by authors. The interpretation introduces a further level of subjectivity and may even include a wish to see a hypothesis “proven.” A publication is an important narrative of the work done and interpretations made by researchers securing a scientific discovery. As The Royal Society neatly states though, Nullius in verba (Take nobody's word for it), whereby the role of the underpinning data is paramount. Therefore, the objectivity that preserving that data within the article provides is due to readers being able to check the calculation decisions of the authors.

Previous articles on this theme with several colleagues have addressed the how and what, describing the archiving of raw diffraction datasets (Tanley *et al*., 2013), and the metadata that are essential to be included (Kroon-Batenburg and Helliwell, 2014; Kroon-Batenburg *et al*., 2017) as well as why we should archive raw diffraction data (Helliwell *et al*., 2017). This article provides a summary of recent experiences with archiving raw diffraction data along with highlighting various milestones from the past.

## II. THE ORGANIZATION OF CRYSTALLOGRAPHIC DATA

This year, the Cambridge Structural Database (CSD) reached one million structures, a huge milestone and achievement. The Protein Data Bank (PDB), launched in 1971, this year reached 150 000 macromolecular structures, which is another massive achievement. The IUCr Journals have made a significant contribution to the growth of the CSD, having contributed nearly 90 000 structures; ∼50% of these in Acta Cryst E. The millionth structure to enter the CSD is 1-(7,9-diacetyl-11-methyl-6*H*-azepino [1,2-*a*]indol-6-yl)propan-2-one (CSD Refcode XOPCAJ). Crystallography data as a scientific field of enquiry have therefore secured many and various aspects of being Findable, Accessible, Interoperable, and Reusable (Wilkinson *et al*., 2016) for many decades. Some specific challenges remain as described, for example, with data interoperability between chemical and protein crystallography for metal complexes (Brink and Helliwell, 2019).

## III. IUCr ACTIVITIES ENSURING TRUST IN CRYSTALLOGRAPHIC DATA

In 1991, the Crystallographic Information Framework approach was established, which defined “cif,” a “crystallographic information file” based ontology (for a review of this, see Hall and McMahon, 2016). Before that, IUCr journals published tables of coordinates and structure factors. Also, let us recall that Bragg in his foundational article on the first crystal structures published his raw diffraction images, his Laue diffraction photos from his alkali halide crystals, with which he showed how he deduced the sodium chloride crystal structure, the “first crystal structure” (Bragg, 1913). He emphasized in his retrospective memoir “The Development of X-ray Analysis” (Bragg, 1975) that he could be sure of his deduction because of monochromatic diffraction measurements made on the X-ray spectrometer invented by his father Bragg (Bragg, 1914).

Coming back to the modern era, in 1991, the Crystallographic Information Framework was introduced and is maintained as a standard by IUCr's ComCifs (the Committee for the Maintenance of the CIF Standard) (https://www.iucr.org/resources/cif/comcifs/terms-of-reference). In 1998, “checkCIF” was introduced by IUCr (https://checkcif.iucr.org/) and has been adopted around the world by all journals publishing chemical crystal structures. From 2003, IUCr assisted the PDB to introduce wwPDB validation (the term validation report was introduced in 2010). The macromolecular version of cif is the “mmcif.” The PDB's validation report was another huge step in ensuring structure and data quality standards on which users' trust is based.

In 2011, the IUCr Executive Committee led by then President Professor Dr. Sine Larsen instigated a formal review of raw diffraction data deposition; the Diffraction Data Deposition Working Group (DDDWG) was setup. After its final report was submitted and approved by the IUCr Executive Committee in August 2017 (https://www.iucr.org/resources/data/dddwg/final-report), the IUCr established a Standing Committee on Data (CommDat, https://www.iucr.org/resources/data/commdat), which absorbed the DDDWG remit and extended its coverage. The terms of reference are as follows: CommDat will advise the IUCr Executive Committee on all aspects of data with respect to policy and actions to be taken.

## IV. DEFINITIONS OF WHAT OUR DATA ZOO IS COMPRISED OF

“Crystallographic data” can mean any or all of the following:

1. the raw measurements from our diffraction experiment; the diffraction images,
2. the processed numerical observations, which are our diffraction reflection intensities, and
3. the derived structural information from our molecular model.

For each of these, the detailed metadata, the data about the data, are vital.

## V. CRYSTALLOGRAPHY DATA ARE FACT AND FAIR

Crystallography as a discipline is widely recognized as achieving FACT (van der Aalst *et al.*, 2017) and FAIR (Wilkinson *et al*., 2016). We can cite the following awards and recognition. The Association of Learned and Professional Society Publishers' Award was made in 2006 to IUCr Journals for publication innovation regarding linking of articles with their underpinning data. The CODATA Prize was awarded in 2014 to Professor Sydney R. Hall, “Editor of Acta Cryst Section C.” The award citation states “He devised a universal self-defining text archive and retrieval (STAR) file format that evolved into the Crystallographic Information Framework (CIF), a momentous contribution in the area of data characterization, and well known to structural chemists and biologists in particular as both a data and publications standard. It enables data validation for articles published by IUCr journals.”

More generally, the European Union Report “Turning FAIR Data into Reality” 2018 stated the following:

• The requirement from academic journals that authors provide data in support to their papers has proven to be potentially culture-changing, as has been the case in crystallography.
• Many data standards are maintained by international scientific unions (e.g., the International Union of Crystallography).

The above two bullet points are quotes from https://ec.europa.eu/info/sites/info/files/turning_fair_into_reality_1.pdf

Further efforts of IUCr to ensure crystallographic data are FACT involve leading by example with its own journals so that in Acta Cryst. B, C, E, and IUCrData, the article narrative, the automatic general validation checks (checkcif), and the underpinning data are checked thoroughly by subject specialists (i.e., the specialist referees). Efforts are underway to extend this to biological crystallography (Helliwell, 2018) and indeed all areas of crystallography and structural science publishing (see, e.g., https://www.iucr.org/resources/data/commdat/vienna-workshop). Also, more recently, IUCr Journals have encouraged and expedited citations of the DOIs for the raw diffraction dataset underpinning publications; examples can be found in IUCrJ, Acta Cryst D, and Acta Cryst F. As a future development, priority is being given to a checkcif for raw diffraction data by the IUCr DDDWG and now being taken forward by the IUCr Committee on Data jointly with the Committee for the Maintenance of the CIF Standard (COMCIFS). The PDB and the CSD are both now offering the chance for their data entries to include the DOIs of raw diffraction datasets. Crystallography as a discipline is also striving for our data to be FACT (van der Aalst *et al.*, 2017) and FAIR (Wilkinson *et al*., 2016) now also with Big Data archives, thereby striving for ultimate objectivity. Indeed, this has long been a wish as Strickland *et al*. (2008) remarked: “Ideally, the full scientific record should provide access to the raw data……the IUCr is beginning to consider longer-term approaches to archiving the raw data.”

Why is raw diffraction data archiving important? This is because it allows reuse so as to allow the analysis of the data at higher resolution than used in the original work; it can serve as benchmarks in developing improved methods of analysis, software, and algorithms; it allows checking the interpretation of the symmetries of the crystals; it facilitates analyzing diffraction from multiple lattices present in the crystals; and it makes the analysis of the diffuse scattering that reflects correlated motions or disorder of atoms in the crystals possible.

Note that there is a qualification to my thesis that archiving our raw diffraction data allows us to attain full objectivity. As explained more generally at the Stanford Encyclopedia of Philosophy section on Scientific Objectivity https://plato.stanford.edu/entries/scientific-objectivity/ Sec. 2.3, which it calls “The Experimenter's Regress” (and I paraphrase):

“…reasonable calibration of the instrument takes us from objectivity to a level of subjectivity.”

So our detector calibrations, which we must judge as reasonable, i.e., have been done properly, are required for our raw diffraction data to be deemed acceptable.

Overall, there is the philosophical view of the importance of access to raw diffraction data, namely, analysis through one's own eyes not the lens of someone else. For case studies, see Helliwell *et al*. (2017). The IUCr and CommDat's take-home message is that the IUCr (representing the community of crystallographers and structural scientists) maintains the need for the highest quality of data management at all stages, from experimental data collection, through reduction and analysis, to publication and database deposition.

## VI. MODERN DATA FLOWS IN BIOLOGICAL CRYSTALLOGRAPHY

Modern data flows in biological crystallography are pushing the limits of current handling capacity, and are still accelerating. So, can the wish for realizing scientific objectivity in a publication via its direct link to the archived underpinning primary experimental data, as mentioned in the introduction and shown in Fig. 1, cope with these developments? It is an unanswered question at present for us all in crystallography. Suffice it to say, these increased data flows are deepening and widening the scope of crystallographic science research and applications and are welcome. Synchrotron facilities, in particular, have pushed these data flows up enormously in our field, commencing with the first partly or fully dedicated storage rings in the 1980s. The arrival of the 3rd generation facilities in the 1990s again hugely increased these data flows. The considerable challenges of realizing routinely operating electronic detectors have steadily overcome transitioning from predominantly films in macromolecular crystallography through the stages of the TV intensifier, image plate, CCD, and pixel detector. Dynamic, time-resolved studies, reaching subnanosecond time-resolution synchrotron Laue diffraction and femtosecond serial crystallography with X-ray lasers, are major milestones in scientific opportunities and also have increased data flows. At International Data Week in Denver in 2016, I represented the IUCr and remarked in a session on Big Data archives that the Zenodo Big Data archive was marvelous, including being free to users to deposit. But I added that the maximum dataset size per deposit of a few gigabytes was a serious restriction for time-resolved multiple structural snapshots where one study needed of course each time snapshot to have an archived raw diffraction images dataset. Within a week or two of my getting back from the conference, I noticed Zenodo had increased the allowed data deposit per study to 50 GB!

Another major push is the Coherent diffraction data archive led by Filipe Maia [https://www.cxidb.org/ and Maia (2012)], which allows even terabyte-sized deposits.

There is a wide range of Big Data archiving developments. Others that can be highlighted are as follows: the University of Virginia BD2K for MX, led by Wladek Minor http://www.proteindiffraction.org/(USA) (Grabowski *et al*., 2016) and the Structural Biology Data Grid (Meyer *et al*., 2016). A pioneering step was the Store.Synchrotron initiative in Australia (Meyer *et al*., 2014). Most recently, from the synchrotron family, the initiative of the European Synchrotron Radiation Facility (ESRF) with its data archive and policy (https://www.esrf.eu/datapolicy) of archiving of its measured raw datasets with a registered DOI is being steadily implemented. The neutron facilities such as ISIS (https://www.isis.stfc.ac.uk/Pages/home.aspx) and the Institut Laue Langevin (https://www.ill.eu/) are also very notable for their raw data archiving for a long time.

## VII. THE FOURTEEN RECOMMENDATIONS OF THE IUCR DDDWG

This comprehensive list is worthwhile listing again here:

• the authors should provide a permanent and prominent link from their article to the raw datasets, which underpin their journal publication and associated database deposition of processed diffraction data (e.g., structure factor amplitudes and intensities) and coordinates and which should obey the “FAIR” principles that their raw diffraction datasets should be Findable, Accessible, Interoperable, and Reusable (https://www.force11.org/group/fairgroup/fairprinciples);
• a registered digital object identifier (DOI) should be the persistent identifier of choice (rather than a Uniform Resource Locator, URL) as the most sustainable way to identify and locate a raw diffraction dataset;
• an archive of raw diffraction datasets for currently unsolved crystal structures should be pursued;
• an archive of raw diffraction datasets showing significant diffuse scattering should be pursued;
• workshops for research data management training for the community should continue and be sponsored and organized by the IUCr;
• there should be continued regular checking by the IUCr Executive Committee of the progress of the IUCr Commissions logging of their raw diffraction data metadata;
• archived raw diffraction data should be automatically validated wherever possible via a “checkcif for raw data approach,” and be peer reviewed where necessary, at the minimum to include core metadata: beam center of the diffraction image, wavelength, wavelength bandpass (pink beam case), orientation of all axes, pixel sizes, detector position, and orientations;
• jointly with the IUCr Commission on Crystallographic Computing, the IUCr should pursue reproducibility of science objectives that require open source software and accurate versioning;
• IUCr should engage with vendors and the World Data System to promote the certification of raw diffraction data standards;
• IUCr's CommDat, which first met in Hyderabad in 2017, should continue the directory of data archives by adding any new data archives that are established in future [these are currently listed and described in Kroon-Batenburg *et al.* (2017)];
• IUCr should invite the community to alert CommDat of further case studies that document the value of archiving of raw diffraction data [case studies were published in Helliwell *et al*. (2017)];
• IUCr recognizes that metadata for the sample are clearly vital for all the IUCr Commissions (and are especially diverse in small angle scattering) and whose standardized descriptions should be actively pursued by the Commissions;
• CommDat should regularly monitor the evolution of technology as the pace of change in data measurement rates, and of metadata logging, with new detectors, computer hardware, networks, and electronic laboratory notebooks is especially notable;
• IUCr should actively support the neutron, synchrotron, and X-ray laser facilities in their raw data archiving activities.

Resulting from this report from the IUCr DDDWG, the IUCr Commissions are discussing and acting on them. There are the actions of the “IUCr Commission on Biological Macromolecules” from which an IUCr FAIR policy for raw diffraction data in MX has recently published in IUCrJ, Acta Cryst D, F, and J Appl Cryst (see Helliwell *et al*., 2019). There is the action of the “IUCr Commission on Structural Chemistry” that has launched a survey via the IUCr Newsletter gathering views of its community on the DDDWG's Final Report Recommendations.

## VIII. CONCLUSIONS

Crystallography as a discipline is firmly continuing its traditions of linking data with publication, striving for the very best possible trust in our results. We follow The Royal Society's motto in effect “Don't take our word for it,” because we also provide our data!

On a second aspect, I comment on our entering the era of open science. I note that it is of great interest to policy makers, whose interpretation of this presents all scientists, ourselves as crystallographers included, with the issue that there is expected to be a limited time for funded researchers to analyze their data and publish, typically 3 years. Then, it is expected that the raw data will be put on open access, which is the policy makers' view. A simple first point to make is that where a Ph.D. student's work is involved, this also must have proper account taken of the training periods needed before discovery is made. Suffice to say, new rules of conduct for such funded “open research” will be essential and need to be made crystal clear.

The IUCr also runs a Forum for public inputs on data matters. This is here: https://forums.iucr.org/viewforum.php?f=39.

Once a person has registered themselves, they can post comments, give weblinks to relevant reports, and so on. Alternatively, without logging on, people can browse and download documents already posted there. Since 2011, when this was started by the DDDWG, this forum has obviously served a very useful purpose as judged by the large number of postings and the very large numbers of downloads.
